# Stimulus-Dependent Adjustment of Reward Prediction Error in the Midbrain

**DOI:** 10.1371/journal.pone.0028337

**Published:** 2011-12-02

**Authors:** Hiromasa Takemura, Kazuyuki Samejima, Rufin Vogels, Masamichi Sakagami, Jiro Okuda

**Affiliations:** 1 Department of Life Sciences, The University of Tokyo, Tokyo, Japan; 2 Brain Science Research Center, Tamagawa University Brain Science Institute, Machida, Tokyo, Japan; 3 Laboratorium voor Neuro-en-Psychofysiologie, Katholieke Universiteit Leuven Medical School, Leuven, Belgium; 4 Khoyama Center for Neuroscience, Department of Intelligent Systems, Faculty of Computer Science and Engineering, Kyoto Sangyo University, Kyoto, Japan; Kyushu University, Japan

## Abstract

Previous reports have described that neural activities in midbrain dopamine areas are sensitive to unexpected reward delivery and omission. These activities are correlated with reward prediction error in reinforcement learning models, the difference between predicted reward values and the obtained reward outcome. These findings suggest that the reward prediction error signal in the brain updates reward prediction through stimulus–reward experiences. It remains unknown, however, how sensory processing of reward-predicting stimuli contributes to the computation of reward prediction error. To elucidate this issue, we examined the relation between stimulus discriminability of the reward-predicting stimuli and the reward prediction error signal in the brain using functional magnetic resonance imaging (fMRI). Before main experiments, subjects learned an association between the orientation of a perceptually salient (high-contrast) Gabor patch and a juice reward. The subjects were then presented with lower-contrast Gabor patch stimuli to predict a reward. We calculated the correlation between fMRI signals and reward prediction error in two reinforcement learning models: a model including the modulation of reward prediction by stimulus discriminability and a model excluding this modulation. Results showed that fMRI signals in the midbrain are more highly correlated with reward prediction error in the model that includes stimulus discriminability than in the model that excludes stimulus discriminability. No regions showed higher correlation with the model that excludes stimulus discriminability. Moreover, results show that the difference in correlation between the two models was significant from the first session of the experiment, suggesting that the reward computation in the midbrain was modulated based on stimulus discriminability before learning a new contingency between perceptually ambiguous stimuli and a reward. These results suggest that the human reward system can incorporate the level of the stimulus discriminability flexibly into reward computations by modulating previously acquired reward values for a typical stimulus.

## Introduction

Reward prediction is an important function used by humans and animals to make appropriate decisions in various environments. Humans and animals learn whether the sensory information of incoming stimuli is rewarding or harmful through stimulus–reward experiences. Previous reports have described that reward prediction error (the difference between the predicted reward value and obtained reward outcome) occurs when updating reward prediction associated with sensory stimuli. Schultz and colleagues described that the activity of dopamine neurons in monkey midbrain areas (ventral tegmental area, VTA, and substantia nigra) is correlated strongly with reward prediction error [1], [2], [3], [4]. Human neuroimaging studies have demonstrated that fMRI signals in the midbrain and basal ganglia are correlated with reward prediction error [5], [6], [7], [8]. Computational studies have described these reward prediction error activities using reinforcement learning models such as the Rescorla–Wagner model and the temporal difference (TD) model [2], [7], [9], [10], [11], [12], [13], [14]. These results suggest that the reward prediction error signal is represented in the midbrain dopamine neurons and that it is used for updating the association between reward prediction and sensory stimuli.

However, in the natural world, sensory stimuli are often less distinctive depending on environmental factors (e.g. weather and lighting conditions) than they are under experimental conditions in which sensory information of a stimulus is discrete. In the natural environment, stimuli might be difficult to identify as rewarding or harmful. How do animals calculate reward values in such ambiguous circumstances? One possible strategy is trial-by-trial reinforcement learning by repeated stimulus–reward pairings [15], i.e., learning new associations repeatedly between each condition of stimulus and reward outcomes. Another possible strategy might be stimulus-dependent adjustment of reward values, i.e., modulating already-acquired reward values for a typical stimulus according to the discriminability of incoming stimuli.

Results of previous studies of stimulus processing have suggested that information related to stimulus discriminability is represented quantitatively in sensory cortices [16], [17], [18], [19]. How is such a sensory computation incorporated into the computation of reward prediction error in the brain? Recent electrophysiological studies of primates have revealed that the activities of dopamine neurons are modulated by the discriminability of visual stimuli [20], suggesting that the level of stimulus discriminability is reflected in the reward-predicting activity in the brain. Nevertheless, it remains unclear how such sensory information contributes to reward computation in the human brain.

**Figure 1:**
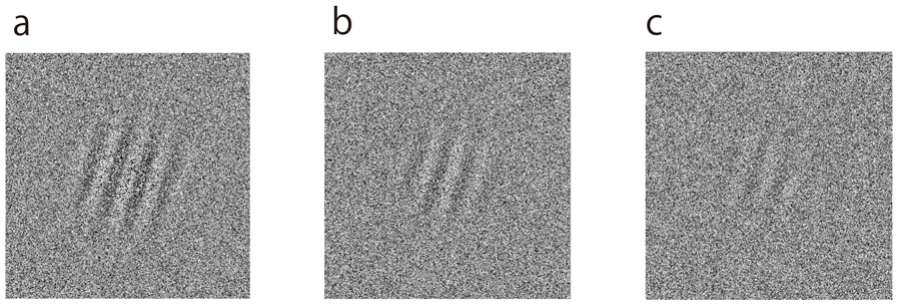
Gabor patch with random dot noise. (a) Stimulus with maximal contrast (99% Michelson contrast) used during conditioning sessions. (b) Typical examples of the stimuli with the contrast corresponding to 90% and (c) 60% correctness of orientation discrimination.

**Figure 2:**
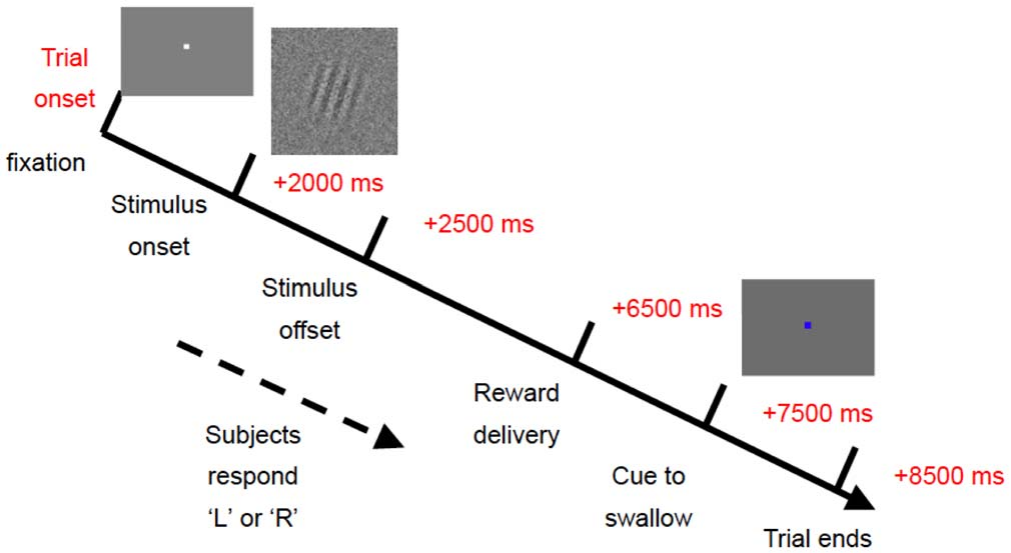
Sequence of one trial. A Gabor patch stimulus was presented to subjects for 500 ms: juice or tasteless saliva was delivered after 4000 ms delay. Subjects were requested to judge the orientation of stimulus as quickly as possible after the stimulus onset. After delivery of the juice or saliva, subjects were allowed to swallow the liquid during presentation of the blue fixation period.

To explore this issue, we investigated the effect of stimulus discriminability on the reward prediction error signal in the human brain by manipulating luminance contrast of a reward-predicting stimulus. We measured the activity of the human brain using fMRI. We used Gabor patch stimuli as reward-predicting stimuli (Figure 1). We defined the stimulus discriminability of Gabor patch based on the orientation discrimination performance of each subject at various luminance contrast levels. We calculated the luminance contrast of the Gabor patch corresponding to 60% and 90% discrimination performance through preliminary psychophysical experiment in each subject (Figure 1). Figure 2 presents the sequence of one trial in the experiment. Subjects learned the contingency between the orientation of the Gabor patch (right or left) and delivery of a juice reward or artificial tasteless saliva in pre-experiment conditioning sessions using maximum (99%) contrast stimuli for which subjects can discriminate orientation almost perfectly (100% discrimination performance). In experimental sessions with fMRI scanning, Gabor patch stimuli with decreased contrast (90% or 60% correctness of orientation discrimination performance for each subject) were presented in a pseudo-random order. To examine the effect of stimulus discriminability on reward prediction error, the orientation-reward contingency was reversed in half of the trials to maximize the number of prediction error trials (unpredicted reward delivery or omission; see Materials and Methods). We examined trial-by-trial variation of brain activities using computational reinforcement learning models (Rescorla–Wagner model) [21].

We used the Rescorla–Wagner rule to evaluate the computational reward prediction error at the time of juice/saliva delivery. We compared two models, one with and one without the factor of stimulus discriminability, to evaluate the relevance of the stimulus information in the computation of reward prediction error (see Materials and Methods). Although our main interest in this study was to explore brain activities related to the reward prediction error signal at the juice/saliva delivery, we also examined brain regions whose activities were correlated with predicted reward values for the Gabor patch stimuli at the time of the stimulus presentation.

## Materials and Methods

### Subjects

In the experiment, 23 healthy normal subjects (8 female, mean age 24.0 years old, SD 4.8) participated. Additionally, six subjects were scanned, but they were excluded from subsequent analyses because of excessive head movements they made during fMRI scanning (>2 mm) or because of their extremely low preference ratings for the juice reward. Two subjects participated only in three experimental sessions (session 1 to session 3) out of four experimental sessions. To maximize the physiological reward value of the juice, subjects were asked to refrain from eating and drinking for 12 hr before the experiment. All subjects gave written informed consent to the experiment. This study, which was approved by the ethical committee of Tamagawa University, followed all Declaration of Helsinki guidelines.

### Stimuli

We used a Gabor patch as the visual stimulus (Figure 1). Stimuli were 11.4×11.4 deg (visual angle). We presented a Gabor patch (*SD* = 1.3 deg; spatial frequency = 0.8 cycles/deg) stimulus at the center of the stimulus area. The Gabor patch was oriented 17 deg to the left or right. We manipulated the luminance contrast of the Gabor patch, and overlaid dynamic random-dot noise patterns to make the left–right orientation judgment sufficiently difficult. Dynamic noise patterns were generated for each frame (refresh rate: 60 Hz) during the presentation of Gabor patch, and overlaid on the whole part of the stimulus. The stimuli were created using Psychophysics Toolbox [22], [23] implemented in Matlab 7 (The MathWorks Inc., USA). The stimuli were backprojected onto a screen located at the end of the MRI magnet bore by a liquid crystalline video-projector. Subjects viewed the stimuli on the screen via a mirror suspended from a head-coil of the MRI scanner. The viewing distance was approximately 60 cm.

We used juice (orange, apple, laichi, or sports drink) as a reward stimulus and tasteless artificial saliva (25 mM KCl and 2.5 mM NaHCO_3_) [24], [25] as a control stimulus. Before the experiment, subjects were asked to evaluate the preference for each juice. We used the juice that the subject most preferred as the reward stimulus in the experiment. The juice and saliva were delivered to the subject's mouth through a plastic tube that ended with a mouthpiece. The amount and timing of the delivery were regulated by an electric solenoid valve that was controlled by a stimulus presentation computer.

### Experimental procedure

The experiment was conducted on two days. On the first day, subjects took part in a psychophysical test and the first conditioning session. On the second day, they participated in the second conditioning session and four experimental sessions. The psychophysical test was performed to measure each subject's psychometric function of the orientation judgment. The subjects were presented 10 different Michelson contrast levels of Gabor patch stimuli and were asked to judge the orientation of the Gabor patch by pressing the corresponding button. Stimuli of each level were presented 20 times in random order. We fitted each subject's behavioral results to the psychometric function of the orientation judgment using Psignifit toolbox (ver. 2.5.6 for Matlab; http://bootstrap-software.org/psignifit/), which implemented the maximum-likelihood method [26]. From the psychometric function, we estimated contrast values corresponding to 90% and 60% correctness for each subject. We used these two contrast values as high (90% correctness) and low (60% correctness) contrast stimuli in the experimental sessions.

In the conditioning and experimental sessions, Gabor patch stimuli were presented for 500 ms with subsequent delivery of juice or saliva after 4000 ms delay. The subjects were asked to judge the orientation of the Gabor patch by pressing a corresponding button as quickly as possible after the Gabor patch onset. Subjects were allowed to drink the liquid stimuli when the color of the central fixation point changed from white to blue (Figure 2).

In the conditioning sessions, a perceptually distinctive Gabor patch (99% Michelson contrast) was used. Subjects were able to discriminate its orientation almost perfectly. To establish conditioning, juice was always associated with one particular orientation and saliva was associated with the other orientation. The delivery of juice or saliva did not depend on each subject's orientation judgment (classical conditioning). The orientation associated with juice was counterbalanced across subjects. Each conditioning session included 20 trials of juice delivery and 20 trials of saliva delivery.

In the experimental sessions, high-contrast and low-contrast Gabor patch stimuli were used. In half of the trials (prediction error trials), juice (or saliva) was delivered after the orientation associated with saliva (or juice) in the conditioning sessions. Therefore, the experimental session consisted of eight conditions (stimulus orientation (left or right)×stimulus contrast (60% or 90%)×liquid delivery (juice or saliva)). Each session consisted of 40 trials (5 trials for each condition) in a pseudo-random order. The inter-trial interval (ITI) was 2 s or 7 s. Because of the presence of ITI of two types, the subjects were unable to predict the time of the onset of the next trial. In the experimental session, the subjects were informed of the possibility of the juice/saliva delivery after an orientation associated with saliva/juice (i.e. prediction error trial). The percentage of the prediction error trials, however, was not told to the subjects. After each conditioning and experimental session, subjects were asked to evaluate their preference for juice and saliva using a scale ranging from −5 to 5, where −5 = most unpleasant, 5 = most pleasant, and 0 = neutral. After the experiment, 22 subjects filled in a questionnaire about impression of the task. In the questionnaire, subjects were asked to estimate how frequently unpredicted reward or saliva delivery occurred (i.e., the percentage of the prediction error trial) during the experimental sessions.

### Imaging procedure

Whole-brain functional imaging data were acquired using an MRI scanner (1.5 T, Magnetom Sonata; Siemens AG, Germany) with T2*-weighted echo planar imaging (EPI) sequence sensitive to blood-oxygenation-level dependent (BOLD) contrast (4-mm-thick slices; 2 mm inter-slice gap; repetition time 2100 ms; echo time 50 ms; 90 deg flip angle; 192 mm field of view; 64×64 matrix). We used a horizontal–coronal oblique slice orientation of 30 deg relative to the anterior–posterior commissure line [27]. During each experimental session, 250 EPI volumes were acquired.

### Image analysis

Image analyses were performed using SPM2 (www.fil.ion.ucl.ac.uk/spm) [28]. Functional images were corrected for different slice acquisition time, spatially realigned to the first volume of each session to correct for head movements, then spatially normalized to a standard EPI template (Montreal Neurological Institute (MNI) reference brain) [29] with a resampled voxel size of 3×3×3 mm. Spatial smoothing was applied using a Gaussian kernel with full-width at half-maximum (FWHM) of 8 mm.

Functional time-series data were then modeled as a two-stage mixed-effects model for statistical inference. In the first stage, four sessions of 250 EPI volumes each were modeled using a subject-specific, fixed effects general linear model (GLM). Five regressors were incorporated into the GLM according to the computational reward prediction error model based on the Rescorla–Wagner rule [21], i.e., two event regressors corresponding to presentation of Gabor patch stimuli and liquid delivery and three parametric regressors corresponding to the predicted reward value *V*(*t*) at the Gabor patch presentation, the reward prediction error *δ*(*t*), and the binomial reward/non-reward effect at the liquid delivery (see “reward prediction error model” below). High-pass temporal filtering with a cut-off value of 128 s was applied when estimating the GLM. The serial auto-correlation of the fMRI time-series data were modeled as an AR(1) model.

In the second stage, contrast images of the five regressors in the first-stage model of the 23 subjects were incorporated into a one-way analysis of variance (ANOVA) model without a constant term. Correction for non-spherically distributed error terms was applied to the estimation of the across-subjects random-effects ANOVA model [30]. We used data from the first to the third experimental sessions because behavioral results suggested maintenance of the conditioning (reward effects on reaction times) only up to the third session (see Results section). We examined the significance of the temporal correlation of the reward prediction error *δ*(*t*) and predicted reward value *V*(*t*) with BOLD signals by testing whether the contrast value for *δ*(*t*) and *V*(*t*) was significantly greater than zero on a voxel-by-voxel basis. The statistical threshold was set as *P*<.05, corrected for the false discovery rate [31] and *P*<.001, uncorrected for multiple comparison for the whole brain analysis. We also examined the temporal correlation of *δ*(*t*) and BOLD signals in each session to examine the session-by-session variation of temporal correlation.

To elucidate the specificity of the BOLD activity to our hypothetical reward prediction error model that incorporated the effect of stimulus discriminability, we constructed two models: with and without the factor of stimulus discriminability. Then we examined the correlation between trial-by-trial variance of model value *δ*(*t*), which describes the reward prediction error at the juice/saliva delivery, and *V*(*t*), which describes the predicted reward value at Gabor patch presentation with the BOLD signals separately for the two models. Statistical models for fMRI regression analyses (the first level GLM for each subject) had the same degrees of freedom across the two reinforcement learning models because they had the same numbers of factors and data samples (brain image data).

**Figure 3:**
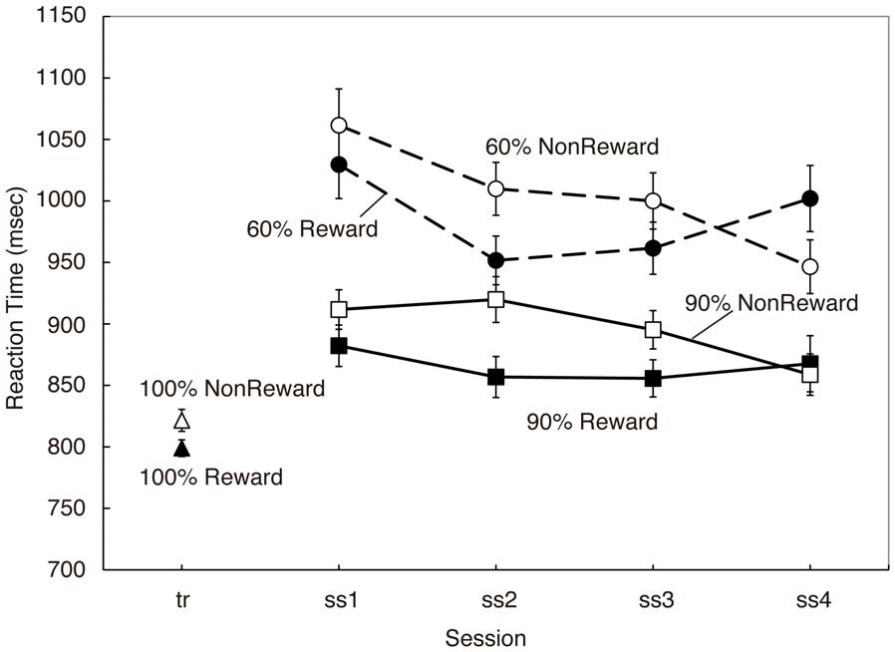
Reaction time results. Reaction time data for each stimulus discriminability, orientation, and session (reward, stimulus conditioned with reward; non-reward, stimulus conditioned with tasteless saliva; tr, average of conditioning sessions; 60%, low-contrast stimuli; 90%, high-contrast stimuli; 100%, maximal contrast stimuli used in conditioning session). Reaction times for stimuli conditioned with reward were significantly shorter than those with non-reward from the conditioning session to experimental session 3. In session 4, this pattern of reaction time difference disappeared. Error bars represent ±1 s.e.m.

Based on the behavioral results showing the reward effect only up to session 3 (Figure 3), we compared the averaged effect sizes from session 1 to session 3 at the peak voxels in areas which showed a significant correlation with *δ*(*t*) at the time of reward delivery or that with *V*(*t*) at the time of Gabor patch presentation (see Table 1 and Table 2, respectively). We compared the effects of *δ*(*t*) and *V*(*t*) between two models using paired *t*-tests without data from the last session (session 4).

**Table 1:** Regions with BOLD responses correlated with reward prediction error values *δ*(*t*) at the time of juice/saliva delivery (*α* = 0.05).

		*X*	*Y*	*Z*	*Z Score*	*Difference between models*
**WITH model**						
Midbrain*		0	−9	−12	5.03	WITH>WITHOUT, *P*<.01
Inferior Frontal Gyrus	Right	30	18	−24	3.68	
Medial Frontal Gyrus	Left	−12	54	18	3.62	
Precentral Gyrus	Right	66	−3	24	5.03	WITH > WITHOUT, *P*<.05
Fusiform Gyrus	Left	−45	−39	−15	3.9	
Inferior Temporal Gyrus	Right	48	−6	−21	3.89	
Parahippocampal Gyrus	Left	−39	−18	−21	3.82	
Thalamus	Right	18	−15	3	4.11	
**WITHOUT model**						
Midbrain		0	−9	−12	4.1	
Precentral Gyrus	Right	66	−3	24	4.35	
Cuneus	Right	9	−78	9	3.38	
Thalamus	Right	18	−15	3	4.2	

Only foci with cluster size >5 are reported.

*, *P*<.05, FDR correction. Other areas were significant at *P*<.001, uncorrected for multiple comparison. The rightmost column shows statistical significance between the WITH model and the WITHOUT model (two-tailed paired *t*-test). WITH>WITHOUT, significantly higher effect size in the WITH model than in the WITHOUT model. WITHOUT>WITH, significantly higher effect size in the WITHOUT model than in the WITH model.

**Table 2:** Regions with BOLD responses correlated with predicted reward values *V*(*t*) at the time of presentation of Gabor patch stimuli (*α* = 0.05).

		*X*	*Y*	*Z*	*Z Score*	*Difference between models*
**WITH model**						
Anterior Cingulate Cortex*	Left	−12	39	6	4.81	
	Left	−9	33	27	3.81	
Superior Frontal Gyrus	Left	−6	12	66	4.04	
	Right	3	0	66	3.72	
Middle Frontal Gyrus	Left	−45	3	39	4.21	
	Left	−48	−33	−6	3.67	WITH>WITHOUT *P*<.05
	Left	−48	21	27	3.49	
	Left	−21	33	48	3.89	
Precentral Gyrus	Right	42	6	30	4.02	WITH>WITHOUT *P*<.05
	Right	48	−6	45	3.87	
Precuneus	Left	−24	−66	39	4	
	Right	21	−60	45	3.58	
Postcentral Gyrus	Left	−12	−45	66	3.92	
	Right	39	−39	60	3.51	
Inferior Parietal Lobule	Left	−48	−39	45	3.52	
Fusiform Gyrus	Right	33	−75	−18	3.38	
Cerebellum	Left	−42	−45	−48	3.88	
	Left	−12	−57	−12	3.63	
	Right	36	−57	−33	4.15	
	Right	24	−54	−36	3.89	
	Right	12	−81	−39	3.66	
	Right	3	−39	0	3.49	
Thalamus	Left	−6	−15	6	3.67	
Putamen/Lateral Globus Pallidus	Left	−15	0	−9	3.49	WITH>WITHOUT *P*<.05
**WITHOUT model**						
Anterior Cingulate Cortex	Left	−12	36	9	3.53	
	Right	9	48	−3	4.24	WITHOUT>WITH *P*<.01
Superior Frontal Gyrus	Left	0	15	54	3.5	
Precentral Gyrus	Left	−39	−9	33	3.53	
	Left	−54	−9	39	3.28	
	Right	57	−3	27	3.63	
Precuneus	Left	−24	−63	39	3.52	
	Left	−6	−66	51	3.36	
	Right	18	−75	33	3.23	
Inferior Parietal Lobule	Left	−45	−39	42	4.4	WITHOUT>WITH *P*<.05
	Left	−54	−30	24	3.6	WITHOUT>WITH *P*<.05
Lingual Gyrus	Left	−6	−63	0	3.6	

Only foci with cluster size >5 are reported.

*, *P*<.05, FDR correction. Other areas were significant at *P*<.001, uncorrected for multiple comparison. The rightmost column shows statistical significance between the WITH model and the WITHOUT model (two-tailed paired *t*-test). WITH>WITHOUT, significantly higher effect size in the WITH model than in the WITHOUT model. WITHOUT>WITH, significantly higher effect size in the WITHOUT model than in the WITH model.

### Reward prediction error model

We adopted the conventional Rescorla–Wagner rule [21] to model the trial-by-trial reward prediction at the Gabor patch presentation and the prediction error at the juice/saliva delivery. In the model, *V*(*t*) represents the reward-prediction value at trial *t*, *r*(*t*) represents the obtained reward value (1 for juice, 0 for tasteless saliva), *α* represents a learning rate, and *δ*(*t*) represents a reward prediction error at the trial *t*. Here, *δ*(*t*) is defined as the difference between the obtained reward *r*(*t*) and the reward-prediction value *V*(*t*).
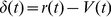
(1)The reward-prediction value at the next trial *V*(*t*+1) is updated based on *V*(*t*) and *δ*(*t*). In the equation below, *α* is the factor of effectiveness of learning (learning rate), ranging from 0 to 1.
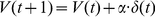
(2)


Reward predictions for stimulus orientations conditioned with reward and those with non-rewarding tasteless saliva in the conditioning sessions were expected to be different. Therefore, in the model simulation, we separately modeled the reward prediction value for an orientation conditioned with a reward as *Vr*(*t*) and that with non-rewarding saliva as *Vn*(*t*). At the first trial of the experimental session, *Vr*(1) and *Vn*(1) were set, respectively, as 1 and 0. For trials in which the subject's judged orientation was not congruent with the stimulus orientation (incorrect trials), we defined *Vr*(*t*) and *Vn*(*t*) based on the judged orientation by subjects, not the orientation of the presented stimulus.

To summarize, in a model that did not consider the factor of stimulus discriminability (hereinafter, WITHOUT model), we calculated the model values based on the formula below.

### (A) WITHOUT model

For trials in which subjects judged the orientation as the reward direction:
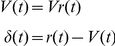



For trials in which subjects judged the orientation as the non-reward direction:
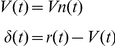



In contrast, in the model that considered modulation by the factor of stimulus discriminability (hereinafter, WITH model), we manipulated the reward-prediction value *V*(*t*) by multiplying the discriminability factor, *p*(*t*) (0.6 or 0.9 for 60% or 90% correctness stimulus, respectively). As shown in the formula below, *V*(*t*) was calculated as a fractional summation of *Vr(t*) and *Vn(t*) multiplied by *p(t*) and (1-*p*(*t*)). The reward prediction value for the orientation relevant to the trial was multiplied by *p*(*t*). That for the irrelevant orientation was multiplied by (1-*p*(*t*)). For the incorrect trials in which judged orientation by the subjects was incongruent with the true stimulus orientation, *p*(*t*) was multiplied on the reward-prediction value for judged orientation and (1-*p*(*t*)) was multiplied on that for the other orientation. The other formula was equivalent to the WITHOUT model.

### (B) WITH model

For trials in which subjects judged the orientation as the reward direction:
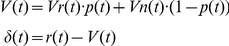



For trials in which subjects judged the orientation as the non-reward direction:
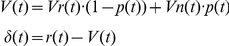



In both models, the predicted reward value in the next trial was modulated by the reward prediction error, *δ*(*t*), as described below.

For trials in which subjects judged the orientation as the reward direction:
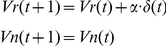



For trials in which subjects judged the orientation as the non-reward direction:
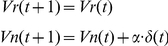



The number of free parameters was equivalent across the WITH model and the WITHOUT model. The common free parameters in the two models were initial values of predicted reward value (*Vr*(1) and *Vn*(1)) and the learning rate (*α*). Given that the learning rate *α* was not known *a priori* [7], we tested eight *α* values for the analysis: *α* = 0.001, 0.005, 0.01, 0.025, 0.05, 0.1, 0.2 and 0.4. We calculated model values (*δ*(*t*) and *V*(*t*)) separately for each learning rate value, and examined temporal correlation between the model values and the brain activity, as measured by fMRI, for each learning rate.

## Results

### Behavioral results

The average correct rate of orientation judgment on the Gabor patch stimuli during the experimental sessions was 89.5% (SD 7.3%) for high-contrast stimuli (equivalent to 90% discrimination performance) and 65.5% (SD 10.1%) for low-contrast stimuli (equivalent to 60% discrimination performance). The correct rate for stimulus orientation conditioned with reward was 91.1% (SD 9.5%) for high-contrast stimuli and 65.4% for low-contrast stimuli (SD 16.5%). The correct rate for stimulus orientation conditioned with tasteless saliva was 88.0% (SD 10.0%) for high-contrast stimuli and 65.6% for low-contrast stimuli (SD 15.1%). The difference of the correct rate between stimulus orientations (conditioned with reward vs. tasteless saliva) was not statistically significant (two-tailed paired *t*-test; *t*(22) = 1.135, *P*>.2 for high-contrast stimuli; *t*(22) = 0.0254, *P*>.2 for low-contrast stimuli).

In contrast, the reaction time was influenced clearly by conditioning with a reward or tasteless saliva. Figure 3 shows reaction time data for each stimulus discriminability, stimulus orientation (conditioned with reward or tasteless saliva) and session. The first five trials of the first experimental session (session 1) were excluded from analyses because reaction times in these trials were significantly longer than those of later trials. Except for the last session, average reaction times for stimuli conditioned with the reward were shorter than those with the tasteless saliva. For statistical analysis, we divided each session into two sub-blocks (the former half trials and the latter half trials). First, to elucidate the reward effect on reaction times in the first three sessions, we examined the effect of conditioning (stimulus orientation) on the reaction time statistically using data up through session 3. A three-way ANOVA (session block (six blocks of the former and latter trials in sessions 1–3), stimulus orientation (reward versus nonreward) and stimulus contrast (60% versus 90%) as within-subject fixed factors and subjects as a random factor) revealed significant main effects of stimulus orientation (*F*(1, 22.2) = 4.89, *P* = .038) and stimulus contrast (*F*(1, 22.2) = 43.36, *P*<.001). Second, we replicated the same analysis for data including session 4. We found a significant main effect of stimulus contrast again (*F*(1, 22.2) = 41.12, *P*<.001), whereas the main effect of stimulus orientation no longer revealed statistical significance (*F*(1, 22.2) = 1.61, *P* = .217). However, a significant interaction was found between the session block and stimulus orientation factors (*F*(7, 150.8) = 2.13, *P* = .044), confirming the effect that the reaction time difference was reversed in the final session (Figure 3). Previous reports have described that reaction times became shorter when the stimulus was conditioned with a reward [32], [33], [34]. Consequently, the shorter reaction times observed for stimuli with reward orientation from sessions 1–3 suggest maintenance of the conditioning up to session 3 and extinction of the conditioning in the final session.

**Figure 4:**
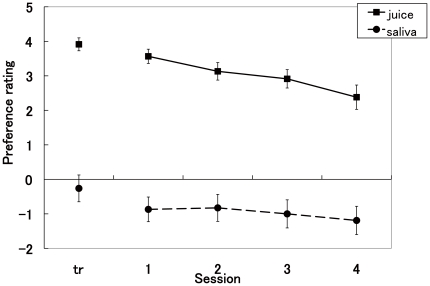
Preference ratings. Rating scores for preference of juice and tasteless saliva in each session (tr: average of conditioning sessions). Error bars represent ±1 s.e.m.

After the completion of each session, subjects rated their preferences for the juice and tasteless saliva. Figure 4 presents results of average preference ratings. Ratings for both juice and saliva decreased in later sessions. Results of a two-way ANOVA (type of stimuli (juice versus saliva), session (conditioning session, experimental sessions 1–4)) revealed significant main effects of the type of stimuli (*F*(1,216) = 359.95, *P*<.001) and session (*F*(4, 216) = 3.69, *P* = .006). Post-hoc paired *t*-tests in each session showed that the ratings for the juice in all sessions were significantly higher than those for the saliva (two-tailed paired *t*-test; *t*(22) = 9.75, 12.34, 8.3, and 8.81 for the conditioning session, experimental sessions 1–3, respectively, and *t*(20) = 7.75 for experimental session 4, *P*<.001 for all sessions, uncorrected for multiple comparison). These results suggest that subjects in all sessions preferred juice to the tasteless saliva.

After the experiment, subjects (excluding one subject) were asked to estimate how frequently the unpredicted reward (or saliva) delivery (reward prediction error trials) occurred in the experimental session. On average, subjects estimated unpredicted reward/saliva delivery occurred in 40.0% of trials (SD = 16.83). This estimated percentage was significantly lower than the actual percentage of reward prediction error trials (50%; two-tailed *t* test, *t*(21) = 2.72, *P*<.05). This result suggests that subjects noticed the presence of reward prediction error trials at some extent, although the estimation of the frequency of reward prediction error trials was lower than the actual one.

### Model simulation results

**Figure 5:**
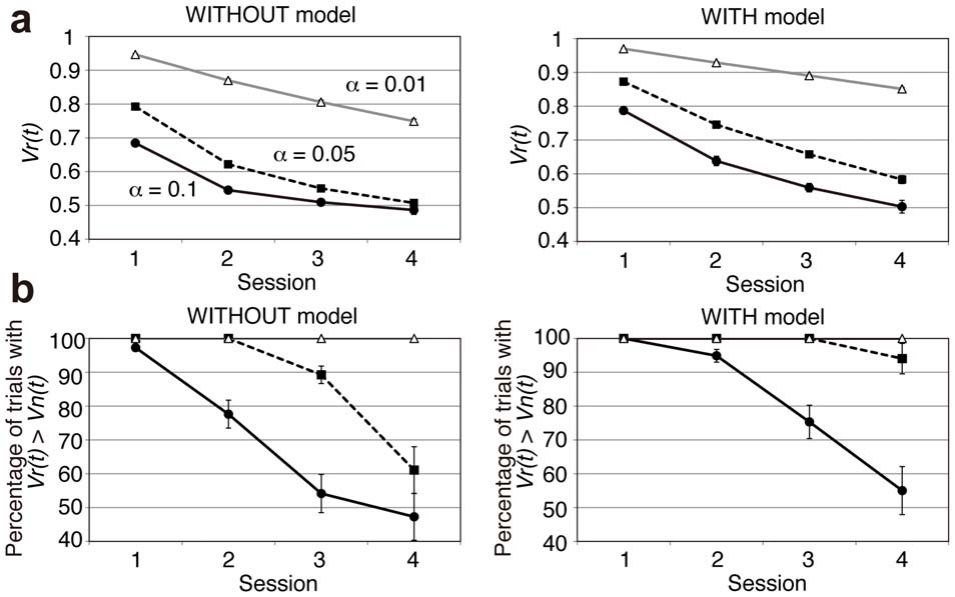
Model simulation of predicted reward values for Gabor patch stimuli. (a) Changes of *Vr*(*t*) and (b) changes in percentage of trials in which *Vr*(*t*) was higher than *Vn*(*t*). Results obtained using three representative values of *α* (0.01, 0.05, and 0.1) are depicted. *Vr*(*t*) decreased faster in the WITHOUT model than WITH model. Error bars represent ±1 s.e.m.

Figure 5a shows changes of predicted reward values for the orientation conditioned with reward (*Vr*(*t*)) for each model with three typical learning rates (*α* = 0.01, 0.05, and 0.1). In the WITHOUT model, *Vr*(*t*) decreased faster than in the WITH model. In both models, *Vr*(*t*) decreased faster with larger learning rates. We examined the percentage of trials in which *Vr*(*t*) was higher than *Vn*(*t*) (predicted reward values for the orientation conditioned with saliva) for each model with the three typical learning rates (Figure 5b). For results obtained using *α* = 0.05, the percentage significantly decreased in session 4, consistent with behavioral results showing the extinction of conditioning at session 4 (Figure 3). However, for the higher learning rate (*α* = 0.1), the percentage approached 50% already at the second session. For lower learning rates (*α* = 0.01), the percentage remained 100% even at the last session.

**Figure 6:**
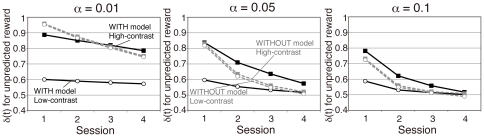
Model simulation of reward prediction error. Changes in *δ*(*t*) for trials in which the unpredicted reward was delivered (positive prediction error trials: subjects judged orientation conditioned with tasteless saliva but juice was delivered). The vertical axis represents the average of *δ*(*t*) for each condition in each session. Results for *α* = 0.01, 0.05 and 0.1 are depicted. Solid black lines represent the WITH model whereas dotted gray lines represent the WITHOUT model. Black squares represent high-contrast (90% correctness) trials whereas open circles represent low-contrast trials (60% correctness). *δ*(*t*) for high and low-contrast stimuli were almost identical in the WITHOUT model, but differed in the WITH model for all learning rates.

Figure 6 shows changes in *δ*(*t*) for trials with unpredicted juice delivery (positive prediction error trials) in two models with three typical learning rates (*α* = 0.01, 0.05, and 0.1). Simulation results of *δ*(*t*) for high-contrast and low-contrast stimuli were quite different in the WITH model, but they were almost identical in the WITHOUT model. Results show that *δ*(*t*) in positive prediction error trials decreased gradually from the first through the last session. In higher learning rates (e.g. *α* = 0.05, and 0.1), *δ*(*t*) became almost equivalent to the minimum value (0.5) in the last session.

### fMRI results

**Correlation with reward prediction error at the time of the juice/saliva delivery:** We calculated the correlation between the fMRI signal at the time of the juice/saliva delivery and trial-by-trial values of reward prediction error, *δ*(*t*), simulated by the two reinforcement learning models (the WITH model and the WIHTOUT model). For both WITH and WITHOUT models, brain regions showing significant correlations with the reward prediction error did not differ greatly across results obtained using different values of the learning rate we tested (8 values from 0.001 to 0.4). In common to both models, the highest correlation between the fMRI signal and reward prediction error was observed in the midbrain region, independently of the learning rate used. These results showing the robust correlation between the fMRI signals in the midbrain region and reward prediction error values were consistent with previous findings that the midbrain encodes the reward prediction error signal in the brain [2], [35].

**Figure 7:**
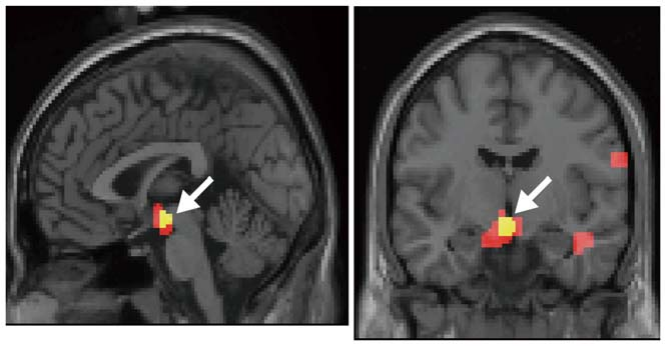
Brain regions showing significant correlation between fMRI signals and reward prediction error values at the time of reward delivery (*n* = 23). A white arrow indicates the midbrain region showing a significant correlation (yellow— *P*<.05, corrected for false discovery rate: red— *P*<.001, uncorrected for multiple comparison) with the variation of prediction error *δ*(*t*) at the time of reward delivery calculated using the WITH model using *α* = 0.05.

Significance for the correlation between the midbrain fMRI signals and the reward prediction error was the highest for the WITH model, *α* = 0.05 (Figure 7, showing the midbrain region correlated with the prediction error at *P*<.05 corrected for the false discovery rate (FDR) [31]; peak MNI coordinate was x = 0, y = −9, z = −12, and peak *Z*-value = 5.03; WITH model, *α* = 0.05). At the threshold of the FDR-corrected *P*<.05, the midbrain correlation was significant only in the result for the WITH model of this learning rate (0.05). Moreover, no region other than the midbrain showed significant correlation at this threshold, even in the result obtained from the WITH model, *α* = 0.05. When we lowered the threshold at *P*<.001, uncorrected for multiple comparison, the correlation in the midbrain was significant also in the results for the WITH model of other learning rates, as well as those for the WITHOUT model of all learning rates. For the results obtained using the learning rate *α* = 0.05 which showed the greatest numbers of regions whose significance was the highest across the eight learning rates, we summarized regions showing significant correlation (both FDR-corrected *P*<.05 and uncorrected *P*<.001) in Table 1, separately for the WITH model and the WITHOUT model. Aside from the midbrain region, we also found a correlation with the reward prediction error in the inferior frontal gyrus, the medial frontal gyrus, the precentral gyrus, the fusiform gyrus, the inferior temporal gyrus, the parahippocampal gyrus, the cuneus, and the thalamus reached a significant level which is uncorrected for multiple comparison (uncorrected *P*<.001).

**Figure 8:**
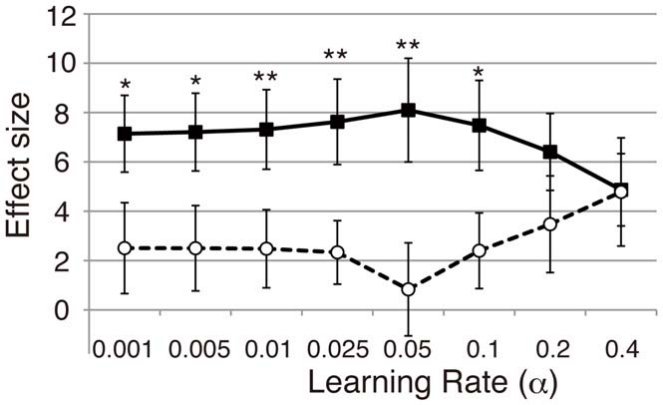
Effect size at the peak midbrain voxel in each learning rate. The effect sizes at the peak midbrain voxel across the eight learning rates are shown for each model (filled squares and thick lines for the WITH model, open circles and dotted lines for the WITHOUT model). Vertical axis represents the effect size (parameter estimates for the regressor of the reward prediction error *δ*(*t*)) at the peak voxel averaged for 23 subjects based on the data up to session 3. The effect size was significantly greater for the WITH model than for the WITHOUT model in most learning rates (two-tailed paired *t*-test: *, *p*<.05; **, *p*<.01). Error bars represent ±1 s.e.m.

We compared the midbrain correlation with the reward prediction error further across models and with different learning rates (*α*). Figure 8 depicts the effect sizes for the regressor component of each model's reward prediction error at the peak midbrain voxel in the eight learning rate (fMRI data up to session 3 were used for the analysis, see the Materials and Methods section). The effect size was significantly higher in the WITH model than in the WITHOUT model in most learning rates (*α* = 0.001, 0.005, 0.01, 0.025, 0.05 and 0.1) except for the higher learning rates (*α* = 0.2 and 0.4). Such higher learning rates were not plausible for the present experimental data according to the fact that model simulation results obtained using such learning rates will not account for behavioral results showing maintenance of the reward effects up to session 3 (Figure 3).

For the midbrain voxel, the difference of the effect size between the models was the highest when *α* = 0.05 (two-tailed paired *t*-test: *t*(22) = 2.91; *P* = .008). Table 1 also shows statistically significant difference in effect sizes between two models (the rightmost column) for each brain regions showing significant correlations with the reward prediction error at this learning rate. Aside from the midbrain region, only the right precentral gyrus (peak coordinate; 66, −3, 24) showed the significantly higher effect size in the WITH model than that in the WITHOUT model (two-tailed paired *t*-test: *t*(22) = 2.44; *P* = .023). However, no area showed significantly higher effect size in the WITHOUT model than in the WITH model.

To summarize the discussion presented above, these fMRI results consistently suggest that the prediction error signal in the midbrain is explained significantly better by considering the factor of discriminability of the reward-predictive stimuli (WITH model). Because the difference of the effect size was observed across most learning rates, the higher correlation in WITH model was independent of the choice of a learning rate (*α*): the results suggested that activities in the midbrain encode prediction-error signals relative to discounted reward-prediction values according to discriminability of the reward-predictive stimuli.

**Figure 9:**
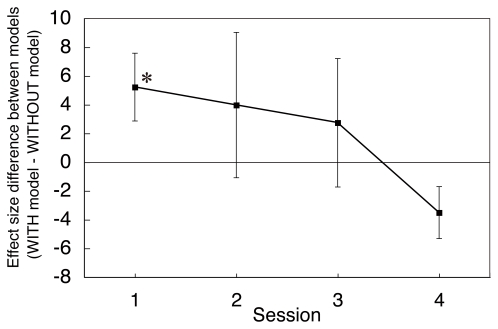
Differences in effect size between the WITH model and WITHOUT model. Positive values represent a greater effect size for the WITH model (*α* = 0.05). An asterisk denotes significant difference between the two models (two-tailed *t*-test: *, *p*<.05). Error bars represent ±1 s.e.m.

Next, we examined session-by-session changes of the correlation between reward prediction error and the midbrain activity to elucidate whether this correlation arises from additional learning between perceptually degraded stimuli and reward/non-reward during the experiment. Figure 9 shows the difference of the significance between the WITH model and the WITHOUT model at the peak midbrain voxel when *α* = 0.05. As shown in this figure, the difference between the models was greatest in the first session (*t*(22) = 2.36, *P* = .03 by two-tailed paired *t*-test of the effect size between the two models). This result suggests that the higher effect size for the correlation with the WITH model is not derived from the new learning, which is expected to result in a smaller correlation in the first experimental sessions. Rather, the data indicate that the activity correlation is instead related to stimulus-dependent modulation of the reward values by the stimulus discriminability from the early phase in the experiment. Consequently, these results indicate that once conditioning between discriminative stimuli and the reward is established, the human reward system can modulate the prediction-error computation adaptively for stimuli of various visibilities before the learning of each stimulus and reward. The difference of the effect size between two models decreased greatly in the final experimental session. This result is also consistent with both behavioral and model simulation data: the reward effect in the behavioral results was diminished in the final session (Figure 3), and model parameter became similar across models in the final session (Figure 6). In addition, reward prediction error values became smaller in the later session in both models (Figure 5), which showed almost equivalent values (0.5) across models in the last session for the learning rates of 0.05 and 0.1.

**Figure 10:**
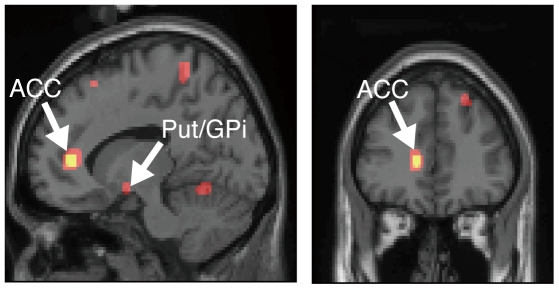
Brain regions showing significant correlation between fMRI signals and predicted reward values at the time of Gabor patch presentation (*n* = 23). White arrows indicate significant correlation with the trial-by-trial variation of the predicted reward values *V*(*t*) calculated using the WITH model, *α* = 0.05 in the left anterior cingulate cortex (ACC; yellow areas — *P*<.05, corrected for false discovery rate) and the left putamen/globus pallidus (Put/GPi, red areas — *P*<.001, uncorrected for multiple comparison). Significant correlations (uncorrected *P*<.001) in several other cortical areas and in the cerebellum were also depicted.

**Correlation with the predicted reward value at the time of Gabor patch presentation:** We also calculated the correlation between the fMRI signal and predicted reward values (*V*(*t*)) in two models at the time of stimulus (Gabor patch) presentation. We only report typical results for the models using a learning rate of 0.05, which revealed the correlation regions that included most regions revealed in the results for other learning rates. At the higher threshold of FDR-corrected *P*<.05, the left anterior cingulate cortex (ACC) was significantly correlated with *V*(*t*) only in the WITH model (Figure 10; peak coordinate, −12, 39, 6; peak *Z*-value = 5.03). However, when we lowered the threshold, we found that the activity of the left putamen/lateral globus pallidus, the cerebellum, and many other cortical areas (Table 2) was correlated significantly with *V*(*t*) in the WITH and the WITHOUT models. As shown in the rightmost column of Table 2, several areas including the left middle frontal gyrus, the right precentral gyrus, and the left putamen/globus pallidus showed significantly higher effect size in the WITH model than in the WITHOUT model for the learning rate 0.05. However, the right ACC and the left inferior parietal lobule showed the opposite pattern (higher effect size in the WITHOUT model than in the WITH model, see Table 2). No other area showed significant difference of the effect size between models.

## Discussion

These results demonstrated that the neural activity in the midbrain is correlated significantly with the reward prediction error in the reinforcement learning model including the factor of stimulus discriminability level (WITH model). This correlation was significantly higher than that obtained with a model without the factor of stimulus discriminability (WITHOUT model). Higher correlation with the WITH model was observed consistently for wide range of learning rates we tested, and no area showed higher correlation with the reward prediction error in the WITHOUT model than that with the WITH model. Furthermore, such a difference of correlation between models appeared from the first session of the experiment. Taken together, these results support the view that the human reward system can incorporate a level of discriminability of perceptually degraded stimuli for calculating the reward prediction error, by adaptively modulating already-acquired reward values for distinctive stimuli according to the stimulus discriminability information related to a stimulus-by-stimulus basis.

Previously, Fiorillo and colleagues [36] have described that the activity in the midbrain dopamine neurons reflects the probability with which stimulus was associated with reward delivery. Their results showed that the reward-related activities (reward prediction and reward prediction error) of the dopamine neurons were modulated according to the reward probability of each stimulus. These results suggest that dopamine neurons can modulate the activity depending on the level of predictability of expected rewards based on the reward probability of each stimulus. Considering this probabilistic coding of reward information in the midbrain dopamine neurons reported previously, it is particularly important to discuss theoretical relation between the probabilistic predictability (or uncertainty) of rewards examined in the Fiorillo's study and the modulation effect by stimulus discriminability revealed in this study. We suggest that major difference between the two studies lies in the mode of acquisition of the uncertainty information for predicting reward. In Fiorillo's study, monkeys learned uncertainty in the association between distinctive stimuli and probabilistic reward delivery (e.g. 60% reward probability) through experiencing multiple trials of stimulus-reward associations. Before such repeated experiences, monkeys have no idea about how much probability of reward is attached to a presented stimulus. In other words, reward probability in the Fiorillo's study represented an uncertainty based on statistical properties across large quantities of trial experiences. In contrast, in the present study, the uncertainty for reward information was derived through perceptual decision of the reward-predictive stimuli on a stimulus-by-stimulus basis, which might not have resulted from averaging over multiple quantities of stimulus-reward experiences. In our experiment, subjects learned stimulus–reward contingency using only a perceptually salient stimulus (with maximum luminance contrast). They have never been presented with low-contrast Gabor patches (equivalent to 60% and 90% orientation discrimination performance) before the start of experimental sessions. Nevertheless, the midbrain activity showed the modulation of activity related to reward prediction error, based on the factor of stimulus discriminability from the first session of the experiment. These results suggest that the reward computation in the brain can be modulated adaptively not only by probabilistic reward computation based on multiple stimulus–reward experiences [36] but also by discrimination probability of incoming sensory stimuli [20]: our results show that human reward system can estimate discounted reward values using information obtained through stimulus processes without requiring additional association between degraded stimulus and reward. This type of “level of stimulus discriminability” is another source of uncertainty that is inherent to stimulus processes of each incoming stimulus, not calculated via statistical properties across numerous experiences. This extension to the mechanism for uncertainty-based reward computation is important to understand the adaptive behaviors of humans and animals in natural environments. For instance, we are often confronted with circumstances where visibility of cues for reward is diminished (e.g. a dark night). We must learn the contingency between the perceptually degraded stimuli and reward again if the reward system can not modulate already-acquired reward values for a particular stimulus according to discriminability of the stimulus. Flexible modulation of reward prediction and reward prediction error according to sensory properties of stimuli is optimal for adapting to changing environments. This study is the first human fMRI study showing the flexible modulation of the reward computation in the dopaminergic system based on the level of stimulus discriminability.

Results of this study revealed that the reward prediction error signal in the midbrain can be modulated flexibly by stimulus discriminability. Then, how was the predicted reward value, which is modulated by stimulus discriminability, represented in the brain? Such a discounted reward value for perceptually ambiguous stimulus is necessary information for the computation of modulated reward prediction error signal in the midbrain. Results show that left ACC correlated significantly with predicted reward value at presentation of Gabor patch stimuli in the WITH model (Figure 10). However, lowering of the threshold revealed that the activity in the basal ganglia (Figure 10; putamen/globus pallidus), the cerebellum, and many other cortical areas was correlated significantly with the predicted reward values (Table 2). Several areas including the left putamen/globus pallidus showed significantly higher effect size in the WITH model, although some other areas including the right ACC showed the opposite pattern. Moreover, most areas showed no significant difference of effect size between the two models. From these data, it is difficult to conclude which areas critically represent discounted reward values for perceptually degraded stimuli. Why were the correlated areas with predicted reward values so widely distributed and why were the differences of correlation between models so variable at the time of the stimulus presentation?

A possible reason is that the experimental paradigm was not necessarily optimized for examining the predicted reward value. In this study, we maximized the proportion of prediction error trials (50%) to examine the reward prediction error signal specifically at the time of juice/saliva delivery. This paradigm is expected to be optimal for examining representation of reward prediction error: the difference of reward prediction error between models was clarified because of the large proportion of prediction error trials. In contrast, the predicted reward value, *V*(*t*), decreased faster because of the presence of prediction error trials (Figure 5). It is difficult to find a distinct area that is specifically related to discounted reward value in the WITH model because the predicted reward value became smaller in most trials. A possible extension of this study is the use of an optimal experimental paradigm for examining predicted reward value at stimulus presentation (e.g. smaller proportion of prediction error trials) to examine the representation of discounted reward prediction specifically, based on stimulus discriminability.

Another possible reason is that the correlation with predicted reward value in distributed areas reflects the mixture of several different functions represented in the brain. Some areas might represent the discounted reward value based on the stimulus discriminability of reward-predicting stimulus. Some other areas might represent the function of monitoring one's own perceptual performance [37]. It was possible that such metacognitive information is useful for reward computation. At the time of stimulus onset, subjects would have to use functions of many types to solve perceptually demanding tasks. Dissociating several different functions at the stimulus presentation (reward prediction based on stimulus discriminability, metacognition, and visual attention) requires examination in future investigations.

Finally, the “stimulus discriminability” in this study might not only correspond to purely objective orientation discrimination sensitivity of subjects. In this study, we did not measure subjective confidence on perceptual decision-making, separately from the objective sensitivity of discrimination performance. It is possible that subjective confidence also provides useful information for the modulation of reward prediction error computation in the midbrain. Dissociation between the objective discriminability and subjective confidence [38], [39] is an important extension of this study for additional understanding of the relation between reward computation and perceptual decision-making.

In conclusion, this study demonstrated that the discrimination level of the reward-predictive stimuli can be incorporated adaptively in the reward prediction error computations in the midbrain. These results suggest that the reward system in the human brain can modulate its computation flexibly by receiving information from stimulus processes to adapt efficiently to the changing environment.
